# The effects of strength training versus ski-ergometer training on double-poling capacity of elite junior cross-country skiers

**DOI:** 10.1007/s00421-017-3621-1

**Published:** 2017-06-08

**Authors:** Tomas Carlsson, Lars Wedholm, Johnny Nilsson, Magnus Carlsson

**Affiliations:** School of Education, Health and Social Studies, Dalarna University, Högskolegatan 2, 791 88 Falun, Sweden

**Keywords:** Cross-country skiing, $\dot{V}{\text{O}}_{{ 2 {\text{peak}}}}$, Double poling, Gross efficiency, Maximal speed, Gender differences

## Abstract

**Purpose:**

To compare the effects of strength training versus ski-ergometer training on double-poling gross efficiency (GE), maximal speed (*V*_max_), peak oxygen uptake ($\dot{V}{\text{O}}_{{ 2 {\text{peak}}}}$) for elite male and female junior cross-country skiers.

**Methods:**

Thirty-three elite junior cross-country skiers completed a 6-week training-intervention period with two additional 40-min training sessions per week. The participants were matched in pairs and within each pair randomly assigned to either a strength-training group (STR) or a ski-ergometer-training group (ERG). Before and after the intervention, the participants completed three treadmill roller-skiing tests to determine GE, *V*_max_, and $\dot{V}{\text{O}}_{{ 2 {\text{peak}}}}$. Mixed between-within subjects analysis of variance (ANOVA) was conducted to evaluate differences between and within groups. Paired samples *t* tests were used as post hoc tests to investigate within-group differences.

**Results:**

Both groups improved their *V*_max_ and $\dot{V}{\text{O}}_{{ 2 {\text{peak}}}}$ expressed absolutely (all *P* < 0.01). For the gender-specific sub-groups, it was found that the female skiers in both groups improved both *V*_max_ and $\dot{V}{\text{O}}_{{ 2 {\text{peak}}}}$ expressed absolutely (all *P* < 0.05), whereas the only within-group differences found for the men were improvements of *V*_max_ in the STR group. No between-group differences were found for any of the investigated variables.

**Conclusions:**

Physiological and performance-related variables of importance for skiers were improved for both training regimes. The results demonstrate that the female skiers’ physiological adaptations to training, in general, were greater than those of the men. The magnitude of the physiological adaptations was similar for both training regimes.

## Introduction

During the last decades, double-poling capacity of skiers has been increasing in importance. A large proportion of the propelling forces are generated by the upper-body muscles (Vähäsöyrinki et al. 2008; Stöggl et al. 2011). In particular, in sprint and long-distance races performed using the classical technique, it is common that elite skiers use skis without kick wax and rely solely on force generation from double poling. As a part of this development, cross-country-skiing research has put a large focus on the double-poling technique. Several studies have investigated technique-related aspects of the double-poling motion (Nilsson et al. 2003, 2013; Lindinger and Holmberg 2011; Holmberg et al. 2005). Others have analysed the physiological demands related to the double-poling technique (Mikkola et al. 2013; Stöggl et al. 2013), and it has been concluded that double poling is more economical than the diagonal stride technique in flat and moderately uphill terrain (Hoffman and Clifford 1992). Moreover, the relationship between physiological abilities of the upper body and performances in cross-country skiing (such as time-trials, ski-rankings, and results from competitions) have been investigated. From an energy-supply perspective, it is important to be able to effectively use metabolic energy in the force-generating muscles in the upper body. This is supported by correlations between elite skiers’ performance capacity and peak oxygen uptake using the double-poling technique ($\dot{V}{\text{O}}_{{ 2 {\text{peak}}}}$) (Carlsson et al. 2012, 2014; Mahood et al. 2001; Sandbakk et al. 2016). It has been suggested that gross efficiency using double-poling technique (GE) (i.e. the ratio between mechanical and metabolic rates) is the strongest predictor of sprint performance (Andersson et al. 2016). Furthermore, ski-specific upper-body power production, measured as maximal double-poling speed (*V*_max_), is also of importance for performance in cross-country skiing (Andersson et al. 2010; Carlsson et al. 2016).

Several studies have investigated the effect of different training regimes, such as strength training (Hoff et al. 1999, 2002; Losnegard et al. 2011; Mikkola et al. 2007; Nesser et al. 2004; Paavolainen et al. 1991; Skattebo et al. 2016; Østerås et al. 2002) and ski-specific training (i.e. double-poling training on ski-ergometer or roller skis) (Downing and Wilcox 2003; Mikkola et al. 2007; Nilsson et al. 2004; Terzis et al. 2006) on the development of skiers’ double-poling capacity. Improvement of $\dot{V}{\text{O}}_{{ 2 {\text{peak}}}}$ in elite skiers has been found after a 6-week training period with 3-min intervals on a ski-ergometer (Nilsson et al. 2004), whereas no change in $\dot{V}{\text{O}}_{{ 2 {\text{peak}}}}$ compared to controls was shown as a result of strength training (Hoff et al. 1999; Mikkola et al. 2007; Skattebo et al. 2016) or short (10–20 s) double-poling intervals (Mikkola et al. 2007; Nilsson et al. 2004). The ability to generate a high *V*_max_ was improved subsequent to an intervention with combined strength training and ski-specific training (Mikkola et al. 2007). A better work efficiency or a lower oxygen consumption using double-poling technique at a constant sub-maximal work intensity was found as a result of both training regimes (Hoff et al. 1999, 2002; Mikkola et al. 2007; Nilsson et al. 2004; Østerås et al. 2002). In contrast, no significant reduction of oxygen cost was found after upper-body strength training in a group of elite female junior skiers (Skattebo et al. 2016).

The rationale behind the choice of training intervention in this context is based on research suggesting that strength and neuromuscular adaptation of the force-generating musculature is a key factor to improve the double-poling capacity (Nesser et al. 2004; Stöggl et al. 2011). A biomechanical study, based on electromyography analyses, demonstrated that upper-body musculature, such as *m. teres major*, *m. latissimus dorsi*, *m. triceps brachii*, *m. rectus abdominis*, and *m. obliquus externus abdominis*, have a high EMG activity at a double-poling intensity corresponding to 85% of *V*_max_ (Holmberg et al. 2005). In line with heavy strength training that gives strength adaptation in upper-body musculature (Rønnestad et al. 2012), ski-ergometer training with high-intensity intervals has been shown to induce a high relative activation of relevant double-poling musculature (Nilsson 2008), which is high enough to induce an increase in muscle strength (McDonagh and Davies 1984). In line with this, an increased force generation during double poling has been found subsequent to a 6-week period of interval training using a ski ergometer (Nilsson et al. 2004).

Besides the previously mentioned non-significant effect of strength training on $\dot{V}{\text{O}}_{{ 2 {\text{peak}}}}$ for elite female junior cross-country skiers (Skattebo et al. 2016), no previous study has investigated the effect of either training regime on double-poling GE, $\dot{V}{\text{O}}_{{ 2 {\text{peak}}}}$ or *V*_max_ for elite junior skiers. Furthermore, cross-country skiers frequently use ski-specific ski-ergometer training and/or semi-specific strength training in addition to their regular endurance training. Therefore, it is essential to investigate whether or not there are effect-related differences between the two training regimes for improvement of double-poling capacity for elite junior skiers. The purpose of this study was to compare the effects of strength training versus ski-ergometer training on double-poling GE, *V*_max_, and $\dot{V}{\text{O}}_{{ 2 {\text{peak}}}}$ for elite male and female junior cross-country skiers.

## Methods

### Overall design

In order to investigate the effect of two different training regimes focused on the improvement of cross-country skiers’ double-poling capacity, a pre–post-intervention study was implemented. The participants completed a test battery consisting of three treadmill roller-skiing tests using the double-poling technique to determine their GE, *V*_max_, and $\dot{V}{\text{O}}_{{ 2 {\text{peak}}}}$. In addition, three strength tests were performed. The tests were performed pre and post a 6-week training intervention. The mean interval between pre- and post-tests for the three roller-skiing tests was 59 ± 7 days (mean ± standard deviation), whereas the interval between the strength tests was 49 days. The study lasted from the end of August to the end of October/beginning of November (i.e. late pre-competition-season period).

### Participants

Forty-four junior cross-country skiers (19 women and 25 men) volunteered to participate in the study and completed the pre-tests. All subjects gave their written informed consent to participate in the study. An additional written consent from parents or guardians was collected for subjects under the age of 18 years. The test procedures were performed in accordance with the World Medical Association’s Declaration of Helsinki—Ethical Principles for Medical Research Involving Human Subjects 2008, and the study was approved by the Regional Ethical Review Board, Uppsala, Sweden.

Based on the test results from the pre-tests, stature, body mass, sex, age, and training preferences, the participants were matched in pairs and within each pair randomly assigned to either a strength-training group (STR) or a ski-ergometer-training group (ERG). During the training intervention, two specific training sessions per week were added to the participants’ regular training programmes. The participants had to complete at least 9 of 12 specific training sessions to be accepted as sufficient.

Seventy-five percent of the participants (17 women and 16 men) fulfilled the specific training-session criteria and completed all tests included in the study. Descriptive characteristics (Table 1) and test results are based on the 33 participants that completed the study (STR: seven women and seven men; ERG: ten women and nine men).

**Table 1:** Characteristics of the elite junior cross-country skiers in the gender-specific sub-groups

	STR ♂ (*n* = 7)	ERG ♂ (*n* = 9)	STR ♀ (*n* = 7)	ERG ♀ (*n* = 10)
Age (years)	18.5 ± 0.8	17.7 ± 0.9	18.4 ± 1.0	18.4 ± 0.9
Stature (cm)	180.5 ± 5.4	182.0 ± 7.0	172.8 ± 5.8	171.0 ± 6.8
Body mass (kg)	72.5 ± 6.3	72.3 ± 9.0	65.4 ± 6.3	67.1 ± 7.7
$\dot{V}{\text{O}}_{{ 2 {\text{max}}}}$ (ml·min^−1^)	4800 ± 620	5090 ± 760	3800 ± 260	3670 ± 420
$\dot{V}{\text{O}}_{{ 2 {\text{max}}}}$ (ml·min^−1^·kg^−1^)	65.4 ± 7.1	69.2 ± 4.1	56.9 ± 4.0	54.1 ± 5.3

All values are presented as mean ± standard deviation. The participants were matched and randomly assigned to either a strength-training group (STR) or a ski-ergometer-training group (ERG). ♂, male skiers; ♀, female skiers. The participants’ age, stature, and body mass were collected at the pre-test day, whereas their maximal oxygen uptake using the diagonal-stride technique ($\dot{V}{\text{O}}_{{ 2 {\text{max}}}}$) was analysed within a week after the post-tests

### Testing procedures

Prior to the tests, the participants completed a health-status questionnaire containing questions concerning, for example, injuries, medication, training, diet, and hydration. Stature (Harpenden Stadiometer, Holtain Limited, Crymych, Great Britain) and body mass (Midrics 2, Sartorius AG, Goettingen, Germany) were measured. The three roller-skiing tests were performed on a motor-driven treadmill (Saturn 450/300rs, h/p/cosmos sports & medical GmbH, Nussdorf-Traunstein, Germany). The participants used their own poles with plastic tips (Black plastic tip; LEKI Lenhart GmbH, Kirchheim, Germany) and roller skis (Pro-Ski C2, Sterner Specialfabrik AB, Dala-Järna, Sweden) provided by the laboratory. The rolling-resistance coefficient of the roller skis was determined to be 0.021 by using the negative-inclination-equilibrium method previously described (Carlsson et al. 2016).

Throughout the GE and $\dot{V}{\text{O}}_{{ 2 {\text{peak}}}}$ tests, variables of expired air were continuously analysed using a metabolic cart in mixing-chamber mode (Jaeger Oxycon Pro, Erich Jaeger Gmbh, Hoechberg, Germany), which was calibrated according to the specifications of the manufacturer before each test. After the GE and $\dot{V}{\text{O}}_{{ 2 {\text{peak}}}}$ tests, capillary-blood samples were collected from a fingertip and these were thereafter analysed to determine blood-lactate concentrations (Biosen 5140, EKF-diagnostic GmbH, Barleben, Germany).

### Gross-efficiency test

The test to determine the participants’ double-poling gross efficiency (GE) followed 15 min of warm-up roller skiing using the double-poling technique at a treadmill inclination of 2°. During the first 5 min of the warm-up, the participants skied at a self-selected treadmill speed; thereafter, the treadmill speed was individually adjusted with the aim that the work intensity should correspond to a blood-lactate concentration of approximately 2–3 mmol l^−1^ and a respiratory exchange ratio below 1.0. The adjustment was based on the participants’ rate of perceived exertion and heart rate, and the skiers’ coach made the work-intensity adjustment. The determined treadmill speed was used for the subsequent GE test. The GE test was initiated 15 min after the end of the warm-up.

The participants’ GE using the double-poling technique was determined at a treadmill inclination of 2° and the pre-determined treadmill speed; this treadmill speed/inclination combination was also used at the post-test. The duration of the GE test was 5 min, and the GE was calculated as the ratio between the mechanical work rate (MWR) and metabolic rate (MR) during the last minute of the specified intensity.

The MWR (kJ min^−1^) is the sum of the work against gravity and the work related to overcoming the rolling resistance of the roller skis during the last minute of the test and was calculated in accordance with the formula: MWR = ((*m*_e_ · *g* · [sin α] · *v* + *k*_1_ · *m*_e_ · *g* · [cos *α*] · *μ* · *v*) · *k*_2_) · 1000^−1^, where *m*_e_ is mass of the participant including the mass of the equipment (kg), *g* is an acceleration due to gravity of 9.82 m s^−2^ at the location of the laboratory, α is the treadmill inclination (°), v is the treadmill speed (m s^−1^), *k*_1_ is 1 and corresponds to the ratio between the average force applied to the roller skis during the forward rolling phase in double poling and the force caused by body mass and gravity, *μ* is the rolling-resistance coefficient of 0.021, and k_2_ is a conversion factor of 60 to comprise the work performed the last minute of the 4-min stage (s min^−1^). The MR (kJ min^−1^) was based on the participants’ mean oxygen uptake ($\dot{V}{\text{O}}_{{ 2 {\text{mean}}}}$) and the mean respiratory exchange ratio (RER) during the last minute of the test in accordance with the following formula: MR = *k*_3_ · *V*O_2mean_ · *k*_4_, where *k*_3_ is 3.815 + 1.232 · RER, which describes the energy equivalent of oxygen (kcal l^−1^), and *k*_4_ is 4.186 which converts kcal to kJ (kJ kcal^−1^) (Carlsson et al. 2016).

A capillary-blood sample was collected immediately after the end of the 5-min GE test. One of the male participants in the STR group had a blood-lactate concentration exceeding 4.0 mmol l^−1^ and was therefore excluded from the statistical analyses related to GE. The mean blood-lactate values at the pre- and post-tests were 2.9 mmol l^−1^ and 2.5 mmol l^−1^, respectively.

### Maximal-speed test

The *V*_max_ test using the double-poling technique at a treadmill inclination of 2.0° was initiated subsequent to the GE test and 3 min of passive rest. Prior to the *V*_max_ test, the participants were familiarised with the 10-s initial acceleration phase from 0 km h^−1^ to the treadmill speed of the first 4-s stage, which were 16.0 and 20.0 km h^−1^ for female and male participants, respectively. This familiarisation was followed by a 1-min passive rest period. The test to determine the participants’ *V*_max_ started with the 10-s acceleration phase to the gender-specific treadmill speed, which was kept for 4 s. Thereafter, the speed was increased by 1.0 km h^−1^ every 4 s while maintaining the treadmill inclination of 2.0°. The *V*_max_ test was terminated when the front wheels of the roller skis crossed a fictive line indicated by two pre-set markers located on each side of the treadmill belt. The participant had to complete the 4-s stage to obtain a *V*_max_ equal to the actual treadmill speed. One of the male participants in the ERG group fell during the test and was therefore excluded from the statistical analyses related to *V*_max_.

### Peak oxygen-uptake test

The $\dot{V}{\text{O}}_{{ 2 {\text{peak}}}}$ test using the double-poling technique was preceded by a 10-min rest period (2-min passive rest, 4-min active rest using the diagonal-stride technique (women: 9 km h^−1^/2.0°, men: 10 km h^−1^/2.0°), and 4-min passive rest). During the last minute of passive rest, the participant was reattached to the metabolic cart. The treadmill inclination was 2.0° throughout the test and the initial treadmill speeds of the $\dot{V}{\text{O}}_{{ 2 {\text{peak}}}}$ test were 13.0 and 16.0 km h^−1^ for female and male participants, respectively. For the female participants, the treadmill speed was increased by 0.4 km h^−1^ every 30 s, whereas the corresponding speed increment for the men was 0.5 km h^−1^ every 30 s until volitional exhaustion which was regarded as time to exhaustion (TTEX).

The plateau criterion was used to ensure that the participants achieved their technique-specific peak oxygen uptake and the $\dot{V}{\text{O}}_{{ 2 {\text{peak}}}}$ was defined as 60-s mean oxygen uptake during the plateau phase (Poole et al. 2008). Both the peak oxygen uptake expressed absolutely ($\dot{V}{\text{O}}_{{ 2 {\text{peak}}}}$ abs) and normalised to body mass ($\dot{V}{\text{O}}_{{ 2 {\text{peak}}}}$ rel) were used in the statistical analyses. One of the female participants in the ERG group did not meet the criterion of a plateau and was therefore excluded from the statistical analyses related to $\dot{V}{\text{O}}_{{ 2 {\text{peak}}}}$.

### Strength tests

The participants performed three strength tests (bar-dips, vertical sit-ups, and chin-ups) pre and post the training intervention.

The bar-dips were executed on a parallel bar. For it to be considered a correctly executed bar dip, the participants flexed their arms from straight to 90° in the elbow joint (with the upper arms parallel to the horizontal bars) and extended their arms back to the starting position. Their legs were held in a semi-flexed position throughout the test, and no bouncing or pausing was allowed.

The vertical sit-ups were performed with a starting position hanging upside down with fixed ankles, a knee angle of 90°, and hands folded behind the head. With a continuous movement, the participant flexed the hip joint until the elbows touched the knees and thereafter back to the starting position. No bouncing or pausing was allowed.

The starting position for the chin-ups was hanging with a shoulder-width overhand grip (pronated hand grip) on a pull-up bar. The participant pulled the body up so that the top of the hands were level with the jaw and thereafter back to the starting position with extended arms. The legs were held in a semi-flexed position throughout the test and no movement in a way that increases momentum in the pulling phase of the lift was allowed.

The maximum number of correctly executed repetitions was registered for each of the strength tests. The sum of registered repetitions for the three strength tests was used as a measure of upper-body strength (UBS), which is included in the Swedish Ski Association’s strength-requirements profile for cross-country skiers (Swedish-Ski-Association 2005).

### Training intervention

The participants training during the intervention consisted of two training sessions per week in addition to their regular endurance training. Depending on which intervention group they were assigned to, the pre-specified training sessions were either focused on strength training or ski-ergometer training. All group-specific training sessions were supervised by the participants’ coaches.

After a standardised warm-up, the STR group followed a training protocol consisting of five exercises focusing on strength development: (1) for different training days, the participant was allowed to alternate between five repetitions maximum (RM) of snatches, squats or deadlifts at 60–85% of body mass, (2) 8 RM sit-ups or vertical sit-ups, (3) 6 RM pull-downs, chin-ups or seal row, (4) 6 RM of pushdowns, bar-dips or narrow bench press, (5) 6 RM of belly-backs with or without dumbbells. All five exercises were conducted with two sets per exercise and 1-min rest between sets. Thereafter, the participants completed 5–10 min with different core-stability exercises, which were followed by 90 s of cable crunches, 60 s of pulldowns with a rope, and 30 s of triceps pushdowns with a rope without rest between exercises. After 1-min rest, these three exercises were repeated once. Finally, 3 sets of 45 s of medicine-ball slams (3–5 kg) with 15-s rest between sets were conducted. After the group-specific training session, the STR group performed 20 min of roller skiing using the double-poling technique in the heart-rate intensity zone MIT.

The training protocol for the participants in the ERG group consisted of two additional training sessions per week (TS1 and TS2) performed on a ski-ergometer (ThoraxTrainer Elite, Thorax Trainer ApS, Kokkedal, Denmark). The training sessions started with a 10-min standardised warm-up. The TS1 consisted of 4 sets of 5–8 min double-poling intervals at a work intensity of 90–95% of HR_max_ with a 25% rest period. The longer intervals were followed by a pyramid of 5 shorter double-poling intervals (30, 45, 60, 45, and 30 s) at a work intensity of 95–100% of HR_max_ with a 100% rest period. The TS2 consisted of 3–5 sets of 3-min double-poling intervals at a work intensity of 90–95% of HR_max_ with a 1.5-min rest period. This was followed by 10 shorter double-poling intervals (6 sets of 30 s with a 1-min rest period and 4 sets of 10 s with a 2-min rest period) at a work intensity equivalent to 95–100% of HR_max_. During the training intervention, the ERG group completed two training sessions per week (week 1: TS1 + TS1, week 2: TS2 + TS2, and week 3–6: TS1 + TS2).

The weekly training duration during the period from pre- to post-tests is presented in Table 2. The aerobic endurance training was categorised based on four heart-rate zones: low-intensity training [LIT: 60–75% of maximum heart rate (HR_max_)]; moderate-intensity training (MIT: 75–85% of HR_max_); high-intensity training (HIT: 85–95% of HR_max_); and very-high-intensity training (VHIT >95% of HR_max_).

**Table 2:** The weekly training duration during the 6-week training-intervention period of the elite junior cross-country skiers in the gender-specific sub-groups

	STR ♂ (*n* = 7)	ERG ♂ (*n* = 9)	STR ♀ (*n* = 7)	ERG ♀ (*n* = 10)
LIT (60–75% of HR_max_)	8.5 ± 3.1	8.7 ± 2.7	8.3 ± 1.4	8.7 ± 1.8
MIT (75–85% of HR_max_)	0.6 ± 0.4	0.2 ± 0.2	0.6 ± 0.1	0.1 ± 0.1
HIT (85–95% of HR_max_)	0.9 ± 0.4	1.0 ± 0.2	0.9 ± 0.3	1.1 ± 0.2
VHIT (> 95% of HR_max_)	0.2 ± 0.2	0.5 ± 0.2	0.2 ± 0.1	0.4 ± 0.3
∑ Endurance training	10.2 ± 3.3	10.3 ± 1.6	10.0 ± 2.6	10.3 ± 2.0
Strength training	1.3 ± 0.2	0.4 ± 0.1	1.4 ± 0.1	0.5 ± 0.2

All values are presented as mean ± standard deviation and the training is expressed as training hours per week in the specific category

*STR* strength-training group, *ERG* ski-ergometer-training group, *♂* male skiers, *♀* female skiers, *LIT* low-intensity training, *MIT* moderate-intensity training, *HIT* high-intensity training, *VHIT* very-high-intensity training, *∑* Endurance training, sum of the weekly training duration in the categories LIT to VHIT

### Statistical analyses

Test results are presented as the means and standard deviations. Normality of the physiological variables was assessed by using the Shapiro–Wilk test. To check that there were no group differences between the STR and ERG groups prior to the study, due to the 11 skiers that did not complete the study, independent sample Student’s *t* tests were performed. Mixed between–within subjects analysis of variance (ANOVA) was conducted to evaluate differences between and within groups for the test variables GE, *V*_max_, $\dot{V}{\text{O}}_{{ 2 {\text{peak}}}}$ abs, $\dot{V}{\text{O}}_{{ 2 {\text{peak}}}}$ rel, TTEX, and UBS. Furthermore, paired sample Student’s t-tests were used as post hoc tests to investigate within-group differences. All statistical analyses were assumed to be significant at alpha level 0.05. The statistical analyses were conducted using the IBM SPSS Statistics software, Version 23 (IBM Corporation, Armonk, NY, USA).

## Results

### Test results

Test results for the physiological variables pre and post the 6-week training intervention for the training groups STR and ERG are presented in Table 3. Both training groups increased their BM (STR: 69.0 ± 7.0–70.3 ± 7.6 kg, *t* = 2.70 and *P* = 0.018; ERG: 69.5 ± 8.6–70.6 ± 8.4 kg, *t* = 3.58 and *P* = 0.002) and MWR (STR: 7.4 ± 1.9–7.5 ± 1.9 kJ min^−1^, *t* = 3.73 and *P* = 0.002; ERG: 7.8 ± 1.6–8.0 ± 1.6 kJ min^−1^, *t* = 2.89 and *P* = 0.014), whereas no significant change was found for MR (STR: 46.8 ± 8.9–46.9 ± 9.2 kJ min^−1^, *t* = 0.07 and *P* = 0.946; ERG: 48.0 ± 9.0–47.6 ± 9.6 kJ min^−1^, *t* = −0.55 and *P* = 0.591) during the training intervention.

**Table 3:** Within-group effects of the 6-week training intervention for the training regimes

Variable	All		Women		Men	
	STR	ERG	STR	ERG	STR	ERG
GE						
Pre	16.3 ± 1.0	15.7 ± 1.5	16.0 ± 1.3	15.0 ± 1.4	16.6 ± 0.7	16.6 ± 1.1
Post	16.8 ± 1.1	15.9 ± 1.5	16.7 ± 1.3	15.1 ± 1.5	16.9 ± 0.9	16.8 ± 0.9
*V*_max_						
Pre	26.1 ± 3.6	25.6 ± 4.0	23.1 ± 1.9	22.3 ± 1.7	29.0 ± 3.6	29.3 ± 1.9
Post	27.3 ± 3.3^†^	26.2 ± 3.7^‡^	24.4 ± 2.0^‡^	23.1 ± 1.7^†^	30.1 ± 1.1*	29.6 ± 1.8
$\dot{V}{\text{O}}_{{ 2 {\text{peak}}}}$ abs						
Pre	3710 ± 790	3740 ± 840	3140 ± 380	3030 ± 360	4280 ± 660	4450 ± 490
Post	3870 ± 700^‡^	3920 ± 810^‡^	3360 ± 280*	3270 ± 480*	4370 ± 610	4560 ± 490
$\dot{V}{\text{O}}_{{ 2 {\text{peak}}}}$ rel						
Pre	53.4 ± 8.5	54.1 ± 9.6	47.9 ± 4.4	46.3 ± 6.6	59.0 ± 8.1	61.8 ± 3.1
Post	54.9 ± 7.3	55.7 ± 8.8*	50.0 ± 3.1	48.7 ± 6.5*	59.7 ± 7.1	62.7 ± 4.4
TTEX						
Pre	377 ± 122	386 ± 119	354 ± 92	312 ± 109	403 ± 155	451 ± 88
Post	452 ± 120^†^	438 ± 108^†^	446 ± 106^†^	394 ± 129^‡^	460 ± 146^‡^	477 ± 71
UBS						
Pre	42 ± 21	34 ± 17	28 ± 11	23 ± 19	56 ± 20	42 ± 10
Post	49 ± 20^‡^	36 ± 17*	38 ± 15^‡^	27 ± 21*	60 ± 18	43 ± 9

Values are expressed as mean ± standard deviation for the physiological tests completed pre and post the 6-week training intervention. STR, participants in the strength-training group (♀: *n* = 7, ♂: *n* = 7); ERG, participants in the ski-ergometer group (♀: *n* = 10, ♂: *n* = 9). GE, gross efficiency (%); *V*_max_, maximal speed using the double-poling technique (km h^−1^); $\dot{V}{\text{O}}_{{ 2 {\text{peak}}}}$ abs, absolute peak oxygen uptake using the double-poling technique (ml min^−1^); $\dot{V}{\text{O}}_{{ 2 {\text{peak}}}}$ rel, relative peak oxygen uptake using the double-poling technique (ml min^−1^ kg^−1^); TTEX, time to exhaustion during the $\dot{V}{\text{O}}_{{ 2 {\text{peak}}}}$ test (s); UBS, upper-body strength (repetitions). Paired-sample Student’s *t* tests were used to investigate within-group differences between pre- and post-tests, and the differences are reported as * for *P* < 0.05, ‡ for *P* < 0.01, and † for *P* < 0.001

### Within- and between-group differences

Prior to the 6-week training intervention, there were no differences for the physiological variables that were used to analyse the intervention effects between the STR and ERG groups or between the STR and ERG sub-groups when they were divided with respect to gender (*t* = 1.61–0.01, *P* = 0.13 to 0.99).

The ANOVAs revealed that there were substantial main effects for time (i.e. from pre to post) for *V*_max_ (Wilks’ *λ* = 0.45, *F*_1,31_ = 37.3, *P* < 0.001, partial *η* ^2^ = 0.55), $\dot{V}{\text{O}}_{{ 2 {\text{peak}}}}$ abs (Wilks’ *λ* = 0.64, *F*_1,30_ = 17.1, *P* < 0.001, partial *η* ^2^ = 0.36), $\dot{V}{\text{O}}_{{ 2 {\text{peak}}}}$ rel (Wilks’ *λ* = 0.78, *F*_1,30_ = 8.5, *P* = 0.007, partial *η* ^2^ = 0.22), TTEX (Wilks’ λ = 0.33, *F*_1,28_ = 56.3, *P* < 0.001, partial *η* ^2^ = 0.67), and UBS (Wilks’ λ = 0.60, *F*_1,26_ = 17.7, *P* < 0.001, partial *η* ^2^ = 0.40), whereas no significant effect for time was found for GE (Wilks’ *λ* = 0.88, *F*_1,30_ = 4.1, *P* = 0.051, partial *η* ^2^ = 0.12). The main effect comparing the two training regimes showed that there were no significant between-group differences in effect of training for either of the investigated test variables (*P*-values from 0.110 to 0.948). There were no significant interactions between time and group for any of the variables.

For the STR group, post hoc tests displayed increases in *V*_max_ (*t* = 4.78, *P* < 0.001), $\dot{V}{\text{O}}_{{ 2 {\text{peak}}}}$ abs (*t* = 3.05, *P* = 0.009), TTEX (*t* = 6.88, *P* < 0.001), and UBS (*t* = 3.51, *P* = 0.004); corresponding within-group improvements for the ERG group were found for *V*_max_ (*t* = 3.39, *P* = 0.003), $\dot{V}{\text{O}}_{{ 2 {\text{peak}}}}$ abs (*t* = 3.00, *P* = 0.008), $\dot{V}{\text{O}}_{{ 2 {\text{peak}}}}$ rel (*t* = 2.23, *P* = 0.040), TTEX (*t* = 4.24, *P* < 0.001), and UBS (*t* = 2.70, *P* = 0.018) (Table 3).

### Within- and between-group differences for the women

For the women, substantial main effects for time (i.e. from pre to post) were found for *V*_max_ (Wilks’ *λ* = 0.27, *F*_1,15_ = 40.9, *P* < 0.001, partial *η* ^2^ = 0.73), $\dot{V}{\text{O}}_{{ 2 {\text{peak}}}}$ abs (Wilks’ *λ* = 0.51, *F*_1,14_ = 13.7, *P* = 0.002, partial *η* ^2^ = 0.49), $\dot{V}{\text{O}}_{{ 2 {\text{peak}}}}$ rel (Wilks’ *λ* = 0.61, *F*_1,14_ = 9.0, *P* = 0.010, partial *η* ^2^ = 0.39), TTEX (Wilks’ *λ* = 0.20, *F*_1,13_ = 51.8, *P* < 0.001, partial *η* ^2^ = 0.80), and UBS (Wilks’ *λ* = 0.30, *F*_1,11_ = 25.2, *P* < 0.001, partial *η* ^2^ = 0.70), whereas no significant effect for time was found for GE (Wilks’ *λ* = 0.81, *F*_1,15_ = 3.5, *P* = 0.080, partial *η* ^2^ = 0.19). No significant between-group differences were found for any of the investigated variables (*P* values from 0.055 to 0.604). Furthermore, there are no significant interactions between time and group for any of the variables.

The post hoc tests for the female skiers in the STR group showed improvements in *V*_max_ (*t* = 3.94, *P* = 0.008), $\dot{V}{\text{O}}_{{ 2 {\text{peak}}}}$ abs (*t* = 2.88, *P* = 0.028), TTEX (*t* = 6.00, *P* < 0.001), UBS (*t* = 4.11 and *P* = 0.006). Moreover, within-group differences for the female skiers in the ERG group were found for *V*_max_ (*t* = 5.21, *P* < 0.001), $\dot{V}{\text{O}}_{{ 2 {\text{peak}}}}$ abs (*t* = 2.62, *P* = 0.031), $\dot{V}{\text{O}}_{{ 2 {\text{peak}}}}$ rel (*t* = 2.40, *P* = 0.043), TTEX (*t* = 4.51, *P* = 0.003), UBS (*t* = 3.66, *P* = 0.015) (Table 3).

### Within- and between-group differences for the men

For the men, substantial main effects for time (i.e. from pre to post) were found for *V*_max_ (Wilks’ *λ* = 0.63, *F*_1,14_ = 8.2, *P* = 0.013, partial *η* ^2^ = 0.37) and TTEX (Wilks’ *λ* = 0.37, *F*_1,13_ = 21.8, *P* < 0.001, partial *η* ^2^ = 0.63), whereas no significant effect for time was found for either GE (Wilks’ *λ* = 0.93, *F*_1,13_ = 0.9, *P* = 0.353, partial *η* ^2^ = 0.07), $\dot{V}{\text{O}}_{{ 2 {\text{peak}}}}$ abs (Wilks’ *λ* = 0.77, *F*_1,14_ = 4.2, *P* = 0.059, partial *η* ^2^ = 0.23), $\dot{V}{\text{O}}_{{ 2 {\text{peak}}}}$ rel (Wilks’ *λ* = 0.92, *F*_1,14_ = 1.2, *P* = 0.292, partial *η* ^2^ = 0.08), or UBS (Wilks’ *λ* = 0.82, *F*_1,13_ = 2.8, *P* = 0.115, partial *η* ^2^ = 0.18). No significant between-group differences were found for any of the investigated variables. Furthermore, there are no significant interactions between time and group for any of the variables (*P* values from 0.057 to 0.849).

The post hoc tests showed that male skiers in the STR group improved their *V*_max_ (*t* = 2.73, *P* = 0.034) and TTEX (*t* = 4.53, *P* = 0.007), whereas no significant within-group differences were found for the men in the ERG group (Table 3).

## Discussion

The results of this study demonstrate that elite junior cross-country skiers improve their double-poling capacity after a 6-week training intervention independent of additional training type i.e. strength training or ski-ergometer training. Both groups increased their aerobic power and maximal speed using the double-poling technique; however, no significant between-group differences were found for any of the variables. Moreover, the results suggest that the female skiers’ physiological adaptations to training, in general, were larger than the men’s adaptations. Female skiers in both groups improved *V*_max_ and $\dot{V}{\text{O}}_{{ 2 {\text{peak}}}}$ abs, whereas the only within-group difference found for the men was improvements in *V*_max_ in the STR group.

Previously, improvement of *V*_max_ was found after a period of combined strength training and ski-specific training in a group of elite male skiers (Mikkola et al. 2007). However, no previous study has investigated the effect of training on *V*_max_ for elite junior skiers. In the current study, both training groups improved their *V*_max_; hence, to be able to generate a higher maximal double-poling speed, a concomitant increase of the upper-body power production is a prerequisite. Positive training-intervention effects regarding upper-body power production have been shown after strength training (Østerås et al. 2002) and ski-specific training (Downing and Wilcox 2003; Nilsson et al. 2004) for both genders as well as skiers classified as elite and non-elite. Improvement of upper-body power production was also established after a 10-week strength-training intervention for a group of combined male and female junior skiers compared to controls, whereas no gender difference in power-production improvement was found (Nesser et al. 2004).

From a physical point of view, a key factor for generation of a high *V*_max_ is the ability to produce high propelling-force impulses at a high skiing speed. This is emphasised by the reported relationship between pole-force impulse and *V*_max_ (Stöggl et al. 2011). Furthermore, it has been shown that when speed increases toward *V*_max_, the time to peak pole force decreases gradually (Lindinger et al. 2009). In other studies, strength training is reported to reduce skiers’ time to peak pole force (Hoff et al. 1999, 2002; Mikkola et al. 2007). Together, this suggests that strength training might improve *V*_max_, which is in line with the results presented herein.

In the current study, both ski-ergometer training and strength training improved $\dot{V}{\text{O}}_{{ 2 {\text{peak}}}}$ abs, but no difference in intervention effect was found between the training regimes. Previously, it was reported that ski-ergometer training consisting of 3-min intervals gave an improved $\dot{V}{\text{O}}_{{ 2 {\text{peak}}}}$ in a mixed group of male and female skiers, whereas the training group assigned to 20-s intervals did not display any significant effect on $\dot{V}{\text{O}}_{{ 2 {\text{peak}}}}$ (Nilsson et al. 2004); however, the effect of gender on the improvement of $\dot{V}{\text{O}}_{{ 2 {\text{peak}}}}$ was not investigated. The results in the current study show that there is a gender difference where the female skiers in both groups increased their $\dot{V}{\text{O}}_{{ 2 {\text{peak}}}}$ abs, whereas no improvement was found for the male skiers in either group. The lack of effect on $\dot{V}{\text{O}}_{{ 2 {\text{peak}}}}$ has been established for elite male skiers subsequent to interventions with strength training (Hoff et al. 2002) as well as with combined strength training and ski-specific training (Mikkola et al. 2007). In contrast to the positive intervention effect found for the elite junior female skiers in the current study, two previous strength-training interventions could not establish any effect on $\dot{V}{\text{O}}_{{ 2 {\text{peak}}}}$ for female junior skiers (Hoff et al. 1999; Skattebo et al. 2016). These differences in effect of training can partially be explained by training-regimes and performance-level differences. The strength training in one of the studies consisted of only one exercise (3 sets of 6 RM) performed three times per week for nine weeks (Hoff et al. 1999), and the training volume could potentially be too low to reach the physiological adaptation necessary to increase $\dot{V}{\text{O}}_{{ 2 {\text{peak}}}}$. This might also be the case in the above-mentioned study, which reported a weekly strength-training volume and intensity that were comparable to the training regimen in the current study (Skattebo et al. 2016). However, the performance level in terms of $\dot{V}{\text{O}}_{{ 2 {\text{peak}}}}$ rel was somewhat higher than in the current study (54 versus 47 ml min^−1^ kg^−1^), which to some extent could explain why they did not reach a significant $\dot{V}{\text{O}}_{{ 2 {\text{peak}}}}$ improvement (*P* < 0.1) as a result of the intervention (Skattebo et al. 2016).

The 6-week training intervention had a positive effect for most of the evaluated physiological variables; however, no significant improvements of GE were found for either the STR group or the ERG group. This is in line with results in a previous study that found no significant changes in submaximal oxygen cost in either the strength-training group or the control group (Skattebo et al. 2016). In contrast, a previous study that investigated the effect of strength training for female junior skiers found a reduction in oxygen uptake for a submaximal work intensity using the double-poling technique in the strength-training group (Hoff et al. 1999). Although no significant improvements were found in the current study, a couple of studies have showed that elite male and female skiers can improve their double-poling economy as an effect of strength training (Hoff et al. 2002; Mikkola et al. 2007; Østerås et al. 2002) and ski-specific training (Nilsson et al. 2004). Therefore, further investigations are needed to establish which training regime (including e.g. training volume, load, and intensity) that optimally can improve GE in elite junior skiers.

## Conclusions

Both training regimes improved the skiers’ $\dot{V}{\text{O}}_{{ 2 {\text{peak}}}}$ abs and *V*_max_, which are variables related to performance in cross-country skiing. However, no significant improvements were found for GE in either group. Moreover, the results demonstrate that the female skiers’ physiological adaptations to training, in general, were greater than those of the men. The female skiers in both groups improved *V*_max_ and $\dot{V}{\text{O}}_{{ 2 {\text{peak}}}}$ abs, whereas the only within-group difference found for the men was improvements in *V*_max_ in the STR group. No between-group differences were found for the investigated variables; hence, the magnitude of the physiological adaptations is similar for both training regimes.

## Abbreviations

ANOVA: Analysis of variance
GE: Gross efficiency
ERG: Ski-ergometer-training group
HIT: High-intensity training with a heart rate between 85 and 95% of HR_max_
HR_max_: Maximum heart rate
LIT: Low-intensity training with a heart rate between 60 and 75% of HR_max_
MIT: Moderate-intensity training with a heart rate between 75 and 85% of HR_max_
MR: Metabolic rate
MWR: Mechanical work rate
RM: Repetition maximum
STR: Strength-training group
TS1: Training session number 1 for the ERG group
TS2: Training session number 2 for the ERG group
TTEX: Time to exhaustion
UBS: The sum of the repetitions in bar-dips, vertical sit-ups, chin-ups
VHIT: Very-high-intensity training with a heart rate above 95% of HR_max_
*V*_max_: Maximal speed using the double-poling technique
$\dot{V}{\text{O}}_{{ 2 {\text{max}}}}$: Maximal oxygen uptake using the diagonal-stride technique
$\dot{V}{\text{O}}_{{ 2 {\text{peak}}}}$: Peak oxygen uptake using the double-poling technique
$\dot{V}{\text{O}}_{{ 2 {\text{peak}}}}$ abs: Peak oxygen uptake using the double-poling technique expressed absolutely
$\dot{V}{\text{O}}_{{ 2 {\text{peak}}}}$ rel: Peak oxygen uptake using the double-poling technique normalized to body mass
